# Adherence, reliability, and variability of home spirometry telemonitoring in cystic fibrosis

**DOI:** 10.3389/fped.2023.1111088

**Published:** 2023-02-23

**Authors:** Fabien Beaufils, Raphaël Enaud, François Gallode, Grégory Boucher, Julie Macey, Patrick Berger, Michael Fayon, Stéphanie Bui

**Affiliations:** ^1^ Univ. Bordeaux, Centre de Recherche Cardio-Thoracique de Bordeaux, INSERM U1045, Bordeaux Imaging Center, Bordeaux, France; ^2^ CHU Bordeaux, Département de Pédiatrie, CIC-P 1401, Service d’Anatomopathologie, Service d’Exploration Fonctionnelle Respiratoire, Bordeaux, France; ^3^ INSERM, Centre de Recherche Cardio-Thoracique de Bordeaux, U1045, Centre d’Investigation Clinique (CIC-P 1401), Bordeaux, France

**Keywords:** cystic fibrosis, telemonitoring, spirometry, reliability, adherence - compliance - persistance

## Abstract

**Introduction:**

Forced spirometry is the gold standard to assess lung function, but its accessibility may be limited. By contrast, home spirometry telemonitoring allows a multi-weekly lung function follow-up but its real-life adherence, reliability, and variability according to age have been poorly studied in patients with CF (PwCF). We aimed to compare real-life adherence, reliability and variability of home spirometry between children, teenagers and adults with CF.

**Methods:**

This real-life observational study included PwCF followed for six months in whom lung function (*i.e*, forced expiratory volume maximum in 1 s (FEV1), forced vital capacity (FVC), forced mid-expiratory flow (FEF) and FEV1/FVC ratio) was monitored by both conventional and home spirometry between July 2015 and December 2021. The adherence, reliability and variability of home spirometry was assessed in all PwCF and compared between children (<12years old), teenagers (12–18 years old) and adults.

**Results:**

174 PwCF were included (74 children, 43 teenagers and 57 adults). Home spirometry was used at least one time per week by 64.1 ± 4.9% PwCF, more frequently in children and teenagers than in adults (79.4 ± 2.9%, 69.2 ± 5.5% and 40.4 ± 11.5% respectively). The reliability to conventional lung function testing was good for all assessed parameters (*e.g.*, FEV1: r = 0.91, *p* < 0.01) and the variability over the 6 months of observation was low (FEV1 coefficient of variation = 11.5%). For each parameter, reliability was better, and the variability was lower in adults than in teenagers than in children

**Conclusion:**

Home spirometry telemonitoring appears to be a reliable tool for multi-weekly lung function follow-up of PwCF.

## Introduction

Cystic fibrosis (CF) is one of the most common severe autosomal recessive disease (1). CF is related to mutations in the *cystic fibrosis transmembrane conductance regulator* (CFTR) gene, which encodes the CFTR epithelial ion channel involved in chloride and bicarbonate transport, leading to impaired mucus hydration and clearance (2).

In the lung, CFTR dysfunction increases the risk of pulmonary exacerbation, chronic pulmonary infections and inflammation resulting in increased lung function decline (3–5) and decreased survival (6). Respiratory disease remains the leading cause of death in patients with CF (PwCF) despite CFTR function improvement by the CFTR modulators developed in the last decade (7–10). Thus, proper lung function monitoring remains a major goal in the follow-up of PwCF.

Forced spirometry is the gold standard test to follow lung function (11) by measuring the forced expiratory volume in one second (FEV1), the forced vital capacity (FVC), the forced expiratory flow between 25% and 75% of FVC (FEF25-75), and the FEV1/FVC ratio. Previous studies demonstrated an increased lung function decline in patients with cystic fibrosis (4), the change and the failure to recover the baseline FEV1 after pulmonary exacerbation (3, 5). However, forced spirometry performed in pulmonary function laboratories requires a trained technician and its accessibility can be limited (12).

In recent years, the rise of telemedicine and the development of home spirometry telemonitoring, particularly during COVID-19 pandemic, resulted in monitoring of the patient's lung function several times a week at home. However, the usefulness of these devices requires both good patient compliance and reliable and reproducible results compared to conventional spirometry. Home spirometry telemonitoring devices have already demonstrated a good quality of performed spirometry (13), adherence (14) and a good reliability of home spirometry results compared to lung function results obtained in a dedicated laboratory in a small group of CF patients (14–16). However, most of the studies involving home spirometry included mainly teenagers (12–18 years old) and/or adults (>18 years old) (13, 16–18). The differences in adherence, reliability and variability of home spirometry between children, teenagers and adults with CF have never been assessed previously.

At Bordeaux University Hospital, home spirometry telemonitoring using the Bluetooth-enabled MIR-Spirobank® Smart device has been included in the patient follow-up since 2015, first in children and teenagers and then in adults. We aimed to assess and compare adherence, reliability and variability of home spirometry between children, teenagers and adults with CF.

## Materials and methods

### Study design

This observational, real-life study was conducted at the Bordeaux University Hospital, France. The study was conducted in accordance with the Declaration of Helsinki principles and approved by the South-West and Overseas Protection Committee (CPP) III. According to the law in force in France, the non-opposition of the patient and/or his legal representatives for patients under 18 years of age was obtained for the use of clinical data and lung function testing results which did not require the patients' informed consent.

The study included patients with confirmed CF (sweat chloride >60 mmol/L and/or CFTR gene mutations) followed at the paediatric or adult CF centres older than 5 years old, able to perform forced spirometry and using home spirometry in routine care between July 2015 and June 2021. Patients were included at the first use of the Spirobank smart® and were followed up for 6 months. At the beginning of the follow-up with the Spirobank smart®, patients were advised to perform at least 3 measurements per week for children (6–12 years) and teenagers (12–18 years), and at least one weekly measurement for adults. The advised number of measurements was chosen to fit with the number of respiratory physiotherapy sessions per week performed by patients (i.e., at least 3 times a week for children and at least once for adults). Children were also advised to be helped by their parents to performed home spirometry. The physiotherapists were encouraged to remind and/or to help patients to use the device during respiratory physiotherapy sessions.

### Data collected

At inclusion, we collected clinical data from the patient's medical files including the age, gender, body mass index (BMI), CFTR mutations, comorbidities [pancreatic insufficiency, cystic fibrosis related diabetes (CFRD)], chronic colonization status for pseudomonas aeruginosa (PA) and methicillin susceptible staphylococcus aureus (MSSA), treatments (inhaled bronchodilator, inhaled corticosteroid, CFTR modulators) and results of the last lung function testing (LFT) performed in a dedicated laboratory at the physiology department of the University Hospital of Bordeaux (*i.e*., FEV1, FVC, FEF25-75, and FEV1/FVC).

During the follow-up period, we also collected LFT results from both the first lung function testing performed in our lab after inclusion (named thereafter FEV1_conv_, FVC_conv_, and FEF25-75_conv_, FEV1/FVC_conv_) and results from forced spirometry measurements performed at home with Spirobank smart®. Of note, results from forced spirometry performed with the Spirobank smart® are automatically recorded in the dedicated smartphone application by Bluethooth® at each use. Data recorded by the application (Pneumotel®) are then anonymously transmitted by the patient in real time to the Pneumotel® platform Data are then collected by AquiRespi, another platform dedicated to the coordination of respiratory care in the New Aquitaine region (France) which is in contact with the patients' practitioners. For each patient, all data from home spirometry collected by AquiRespi during the 6 months after inclusion were collected by the investigators (FB, GB). We specifically identified the results of the home spirometry closest to the conventional LFT as FEV1_home M1_, FVC_home M1_, and FEF25-75_home M1_, FEV1/FVC_home M1,_ and those of the second closest home spirometry to conventional LFT as FEV1_home M2_, FVC_home M2_, and FEF25-75_home M2_, FEV1/FVC_home M2_. Patient participation in the study was discontinued if the last recorded measure occurred before the end of the observation period (hereafter called early stoppers).

### Statistical analyses

The analyses were performed using Graphpad Prism 5.1 software (GraphPad Software, La Jolla, CA). Results are presented as absolute values with percentage [*n*/*N* (%)] for categorial variables and as means ± standard deviation (mean ± SD) or median and interquartile ranges (median [IQR_25_; IQR_75_] for quantitative variables.

Adherence was determined by the number of Spirobank smart® uses. Since the recommendations were different for children/teenagers and adults, adherence was assessed by the number of uses normalized by the recommended minimum objective (*i.e.,*; 3 per week for children/teenagers and 1 per week for adults corresponding to 78 and 26 tests for children/teenagers and adults, respectively during the whole follow-up period) and thus expressed as a percentage. Adherence was also analysed using a threshold to identify excellent users (use >80% of the objective rate), good users (use between 50% and 80%), moderate users (use between 30% and 50%) and low users (use < 30%).

Reliability was assessed using Pearson or Spearman correlation tests (Pearson or Spearman tests), Bland and Altman test and intraclass correlation coefficients. Intra-methods agreement was assessed between the two closest measured of conventional LFT. Inter-methods agreement and agreement over time were assessed between conventional LFT and the closest or the second closest home spirometry to the conventional LFT respectively.

We also assessed home spirometry test-to-test variability over time by determining (i) the maximal variability, expressed as absolute value (*Δ*max; *e.g.*, *Δ*max_FEV1 _= Max_FEV1_ - Min_FEV1_) or normalized by the mean of the parameter, (ii) the test-to-test average variability expressed as absolute [*Δ*average; e.g., *Δ*average_FEV1_= (FEV1_n + 1_- FEV1_n_)/n, were n is the number of the tests] or normalized by the mean of the parameter and (iii) the coefficient of variation (CoV; e.g., CoV_FEV1 _= SD_FEV1_/Mean_FEV1_).

Adherence, reliability and variability over time were assessed in the whole population and compared between children, teenagers and adults. Comparisons between groups were performed using Kaplan-Meyer curves and Log-rank test, Fisher's exact test or Chi square test for categorial variables and using were performed using Mann-Whitney or Kruskal-Wallis tests with Dunn post-test for quantitative variables. A *p* value < 0.05 was considered significant.

## Results

### Patient characteristics

The present study included 174 patients composed of 74 children, 43 teenagers and 57 adults with CF whose characteristics are presented in Table 1. Briefly, sex ratio was close to 1/1 and patients were mostly characterized by at least one *Δ*F508 CFTR mutation, pancreatic insufficiency, chronic MSSA colonisation and altered lung function without significant differences between groups (Table 1). CFRD, chronic MSSA colonisation, chronic PA colonisation and treatment by CFTR modulators were significantly more frequent in teenagers and adults than in children (Table 1). Lung function (*i.e.*, FEV1, FVC, FEV1/FVC, FEF25-75 in percentage of predicted values or as Z-score) was significantly decreased in adults compared to both children and teenagers but not significantly different between children and teenagers (Table 1). Except for age, there were no other differences between groups (Table 1).

**Table 1:** Patient characteristics.

Characteristics	All	Children	Teenagers	Adults	*p*
N	174	74	43	57	
Male	86/174 (49.4)	37/74 (50.0)	24/43 (55.8)	25/57 (43.9)	0.492
Age (years)	13.2 [9.6; 20.8]	9.1 [7.6; 10.4]	14.6 [13.1; 16.0]	25.3 [21.3; 33.3]	<0.001^a^^,^^b^^,^^c^
**Mutations**					
Homozygous df508	80/174 (46.0)	35/74 (47.3)	21/43 (48.8)	24/57 (42.1)	0.916
Heterozygous df508	77/174 (44.3)	33/74 (44.6)	18/43 (41.9)	26/57 (45.6)	
Other	17/174 (9.8)	6/74 (8.1)	4/43 (9.3)	7/57 (12.3)	
Pancreatic insufficiency	147/174 (84.5)	63/74 (85.1)	35/43 (81.4)	49/57 (86.0)	0.806
CFRD	31/174 (17.8)	4/74 (5.4)	10/43 (23.3)	17/57 (29.8)	<0.001^a^^,^^b^
**Chronic colonisation**					
*MSSA*	132/174 (75.9)	49/74 (66.2)	40/43 (93.0)	53/57 (93.0)	<0.001^a^^,^^b^
*PA*	63/174 (36.2)	8/74 (10.8)	15/43 (34.9)	40/57 (70.2)	<0.001^a^^,^^b^
**Treatment**					
ICS	85/174 (48.9)	32/74 (43.2)	22/43 (51.2)	31/57 (54.4)	0.423
LABA	96/174 (55.2)	36/74 (48.6)	26/43 (60.5)	34/57 (59.6)	0.329
CFTR modulator	45/174 (25.9)	4/74 (5.4)	16/43 (37.2)	25/57 (43.9)	<0.001^a^^,^^b^
**Last Lung function**					
ppFEV1 (%)	69.1 [56.2; 92.4]	84.0 [63.6; 97.0]	79.2 [61.9; 94.6]	54.1 [42.0; 64.0]	<0.001^b^^,^^c^
FEV1 Z-score	−2.55 [−3.59; −0.63]	−1.40 [−3.08; -0.25]	−1.76 [−3.14; −0.46]	−3.70 [−4.53; −2.69]	<0.001^b^^,^^c^
ppFVC (%)	84.2 [72.1; 98.2]	94.1 [81.4; 102.1]	86.9 [75.8; 99.7]	72.8 [60.7; 83.4]	<0.001^b^^,^^c^
FVC Z-score	−1.37 [−2.39; −0.15]	−0.52 [−1.59; 0.18]	−1.14 [−2.12; −0.03]	−2.29 [−3.30; −1.36]	<0.001^b^^,^^c^
FEV1/FVC (%)	75.3 [63.1; 82.9]	78.9 [70.1; 84.5]	78.1 [72.0; 83.8]	63.0 [57.1; 71.5]	<0.001^b^^,^^c^
FEV1/FVC Z-score	−1.96 [−2.88; −0.87]	−1.53 [−2.40; −0.51]	−1.45 [−2.02; −0.67]	−2.83 [−3.23; −2.21]	<0.001^b^^,^^c^
ppFEF_25−75_ (%)	41.2 [24.9; 77.8]	57.6 [32.6; 84.1]	62.0 [38.9; 80.4]	24.3 [17.9; 31.8]	<0.001^b^^,^^c^
FEF_25−75_ Z-score	−2.92 [−3.92; −1.02]	−1.90 [−3.31; −0.59]	−1.87 [−3.33; −0.93]	−3.97 [−4.59; −3.35]	<0.001^b^^,^^c^

Results are presented as n/N (%) for categorial variables or median with interquartile ranges [median (IQR25; IQR75)] for quantitative variables. Comparison between groups were performed using Chi square test for categorial variables or Kruskal-Wallis test with Dunn post-test for quantitative variables. *p *< 0.05 was considered significant.

^a^ Children vs. teenager.

^b^ Children vs. adults.

^c^ teenagers vs. adults.

BMI, body mass index; CFRD, cystic fibrosis related diabetes; MSSA, staphylococcus aureus methicillin sensitive; PA, pseudomonas aeruginosa; ICS, inhaled corticosteroids; LABA, long-acting beta agonists; FEV1, forced expiratory volume in one second; pp, percentage of predictive values; FVC, force vital capacity; FEF25-75, forced expiratory flow between 25% and 75% of vital capacity.

### Real-life adherence to home spirometry

**Figure 1:**
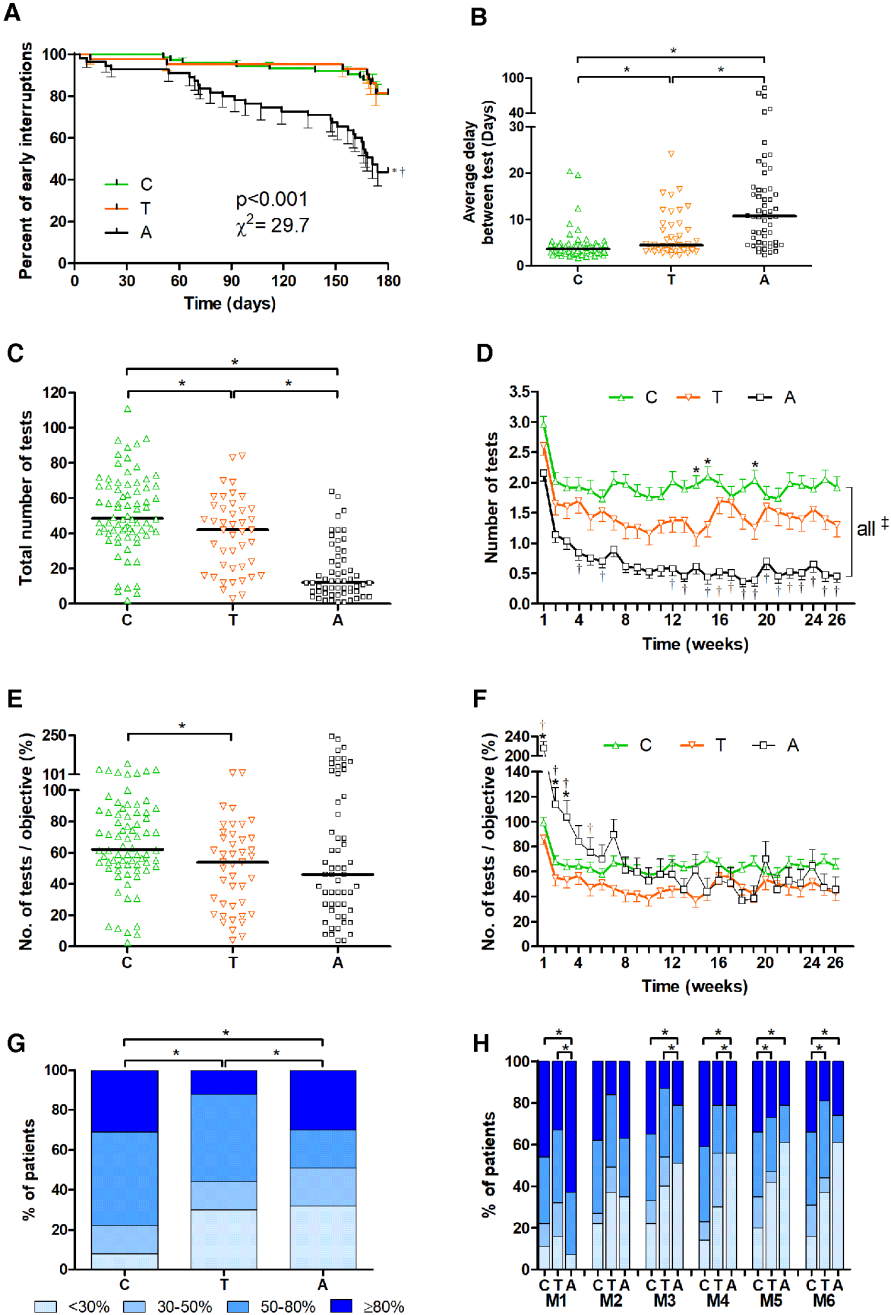
Adherence of home spirometry in the three age groups. Adherence was analysed from home spirometry transmitted data. Comparison between children (6–11 years old; “C”), teenagers (12–17 years old; “T”) and adults (>18 years old; “A”) were performed for the time to early interruption of home spirometry use (**A**), the average test-to-test delay (**B**), the absolute number of tests per patient during the follow-up period (**C**) and the absolute number of tests per patient per week (**D**), the absolute number of tests per patient during the follow-up period normalized by the recommended objective (*i.e.*; 3 per week for children/teenagers and 1 per week for adults corresponding to 78 and 26 tests for children/teenagers and adults respectively during the whole follow-up period) (**E**), the absolute number of tests per patient per week during the follow-up period normalized by the recommended objective (**F**), the percentage of patients with low (achieved <30% of the objective), medium (30%–50%), good (50%–80%) and excellent (>80%) adherence during the follow-up period (**G**) or month-by-month (**H**). Comparisons were performed using Kaplan-Meyer curves and Log-rank test (**A**) or Kruskal-Wallis tests with Dunn post-test for quantitative variables (**B,C,E**) or two-way ANOVA with Bonferroni post-test (**D–F**), Chi square and Fisher exact tests (**G,H**). A *p* value < 0.05 was considered significant.

Within six months after the first use of the Spirobank smart®, 55/174 patients (31.6%) stopped using the device early but the mean time between inclusion and last use during the follow-up period was 162.5 ± 45.0 /180 days. Early stoppers were more often adults than children or teenagers (children 14/74 (18.9%) vs. teenagers 8/43 (18.6%) vs. adults 33/58 (56.9%), *p* < 0.001). Moreover, the time between inclusion and last use during the follow-up period was decreased in adults compared to children or teenagers (134.9 ± 61.1 vs. 171.4 ± 31.1 vs. 171.1 ± 27.6 days respectively; *p* < 0.001). As a consequence, the time to an early stop of the device was significantly shorter in adults than in children or teenagers with no significant difference between the two last groups (Figure 1A). In addition, the average test-to-test delay was 8.5 ± 11.3 in the whole population and significantly increased in adults compared to both children and teenagers and also significantly decreased in children compared to teenagers (Figure 1B).

During the follow-up period, 37.6 ± 24.8 tests/patient corresponding to 1.4 ± 0.3 tests/patient/week were performed in the whole population. Since inclusion started with the first use of the device all patients performed at least one test during the first week of the follow-up period. Then, the percentage of patients with at least one use per week was 64.1 ± 4.9%/week but only 35.1 ± 8.7% of patients/week met the recommended target (*i.e.*, 3 per week for children/teenagers and 1 per week for adults). The total number of home spirometry tests performed was significantly higher in children than in teenagers and adults and in teenagers than in adults (Figure 1C). When the number of home spirometry was analysed week-by-week, it was significantly increased in children compared to teenagers for few weeks (*i.e*., W13, W14, W19) (Figure 1D). Not surprisingly, the number of home spirometry tests performed was lower in adults than in children for all weeks and lower than in teenagers for several weeks (*i.e*., W4, W6, W12, W13, W15 to W26) (Figure 1D). During the whole follow-up period, the percentage of patients with at least one use per week was 79.4 ± 2.9%, 69.2 ± 5.5% and 40.4 ± 11.5% in children, teenagers and adults, respectively.

However, when the total number of tests was normalized by the expected objective (*i.e.,*; 78 tests for children/teenagers and 26 tests and adults during the entire follow-up period), it was significantly increased in children (66.6 ± 28.3%) compared to teenagers (50.2 ± 27.1%) but not different between children and adults (68.7 ± 61.7%) or teenagers and adults (Figure 1E). Moreover, the week-by-week normalized number of tests was significantly increased in adults compared to children and teenagers until the 3rd week of use and until the 5th week of use compared to teenagers and then not significantly different between the three groups (Figure 1F).

During the whole follow-up period, the percentage of excellent, good, moderate, and low users was significantly different between the three groups (Figure 1G). The percentage of excellent users was lower in teenagers than in other groups, that of good users was increased in both children and teenagers compared to adults and the percentage of low users was lower in children than in the other groups (Figure 1G). The percentage of month-by-month of excellent users was increased and that of moderate users was decreased in adults compared to the two other groups in the first month (Figure 1H). In the second month, there was no significant difference between the groups (Figure 1H). Then, from the third to the sixth month the percentage of excellent and good users were significantly increased and that of low users was significantly decreased in children compared to teenagers and adults (Figure 1H).

### Real-life reliability of home spirometry

In our population, 156/174 (89.7%) patients including 67/74 (90.5%) children, 39/43 (90.7%) teenagers and 47/57 (82.4%) adults had conventional LFT between inclusion and their last use during the follow-up. Conventional LFT was performed 74.0 [48.0; 98.0] days after inclusion. The delay between conventional LFT and the closest and the second closest home spirometry test were 1.86 [0.7; 6.11] and 5.2 [2.3; 13.6] days, respectively. The delay between the closest and the second closest home spirometry test was 4.1 [2.0; 10.7] days. There were no significant differences according to these delays between the three groups of age.

**Figure 2:**
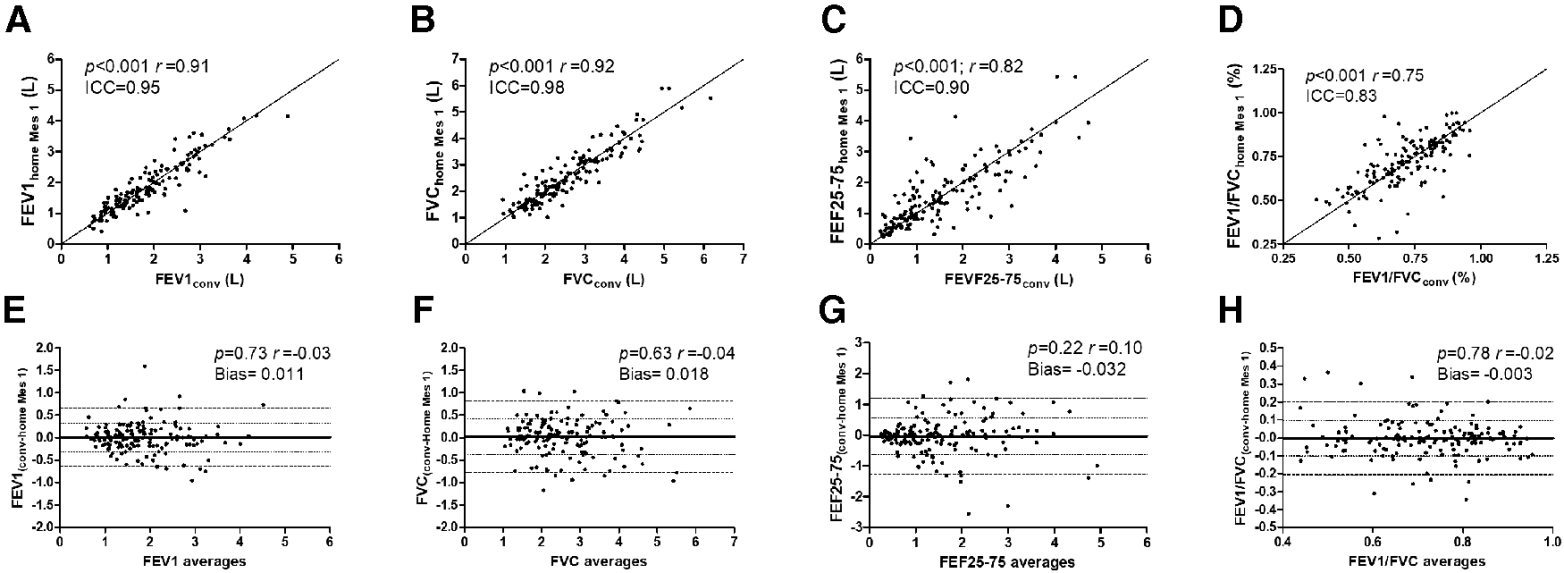
Agreement between conventional and home spirometry results. Agreement between conventional (conv) and its closest home spirometry (Home Mes 1) were assessed using correlation test [the coefficient of correlation *r* and the intraclass correlation (ICC) are presented] (**A–D**), and Bland-Altman test (**E–H**) for the forced expiratory volume in one second (FEV1) (**A,E**), the forced vital capacity (FVC) (**B,F**), the forced expiratory flow between 25 and 75% of FVC (FEF25-75) (**C,G**) or the FEV1/FVC ratio (**D,H**). A *p* value < 0.05 was considered significant.

A Bland-Altman analysis was used to compare lung function results assessed by conventional spirometry and home spirometry. Inter-methods (*i.e.*, conventional compared to 1st closest home spirometry) (Figure 2) and intra-methods agreement (*i.e.*, 1st closest compared to 2nd closest home spirometry) as well as agreement over time (*i.e.*, conventional compared to 2nd closest home spirometry) were excellent for both FEV1 and FVC, good-to-excellent for FEF25-75 and moderate-to-good for FEV1/FVC, as indicated by high correlation coefficients, high intra-class correlation, the lack of correlation in Bland-Altman test and low difference and bias between each two sets of measures (Table 2). When reliability was assessed in each age groups, agreement was systematically better for adults than both teenagers and children for all parameters (*i.e.*, FEV1, FVC, FEF25-75 and FEV1/FVC) and was better in teenagers than in children for FEV1 and FVC but not for FEF25-75 and FEV1/FVC (Table 2). In the whole population and for each group, FEV1 and FVC agreements were always better than FEF25-75 and FEV1/FVC agreements (Table 2).

**Table 2:** Agreements between lung function testing results assessed by both conventional spirometry and home spirometry.

		Inter-method agreement				Intra-methods agreement				Agreement over time			
		All	C	T	A	All	C	T	A	All	C	T	A
*FEV1*	*r*	0.91	0.82	0.83	0.94	0.94	0.84	0.90	0.96	0.91	0.84	0.84	0.92
	*p* value correlation	<0.01	<0.01	<0.01	<0.01	<0.01	<0.01	<0.01	<0.01	<0.01	<0.01	<0.01	<0.01
	Differences	0.0 ± 0.3	0.0 ± 0.3	0.1 ± 0.4	−0.1 ± 0.3	0.0 ± 0.3	0.0 ± 0.3	0.0 ± 0.4	0.0 ± 0.2	0.0 ± 0.3	0.1 ± 0.3	0.1 ± 0.5	−0.1 ± 0.3
	Bland-Altman - *ρ*	−0.03	0.05	0.0	−0.18	−0.07	−0.09	−0.07	0.03	−0.06	−0.03	−0.06	−0.13
	Bland-Altman -*p* value	0.73	0.67	1.00	0.22	0.42	0.46	0.68	0.83	0.47	0.82	0.73	0.36
	Bias	0.01	0.01	0.08	0.08	0.03	0.04	0.03	0.01	0.04	0.07	0.11	−0.06
	ICC	0.95	0.90	0.91	0.97	0.97	0.91	0.95	0.98	0.95	0.91	0.91	0.96
*FVC*	*r*	0.92	0.71	0.89	0.92	0.93	0.71	0.83	0.96	0.93	0.81	0.86	0.93
	*p* value correlation	<0.01	<0.01	<0.01	<0.01	<0.01	<0.01	<0.01	<0.01	<0.01	<0.01	<0.01	<0.01
	Differences	0.0 ± 0.4	0.0 ± 0.4	0.1 ± 0.4	0.0 ± 0.4	0.0 ± 0.4	0.1 ± 0.4	−0.1 ± 0.5	0.0 ± 0.3	0.0 ± 0.4	0.1 ± 0.3	0.0 ± 0.5	0.0 ± 0.4
	Bland-Altman - ρ	−0.04	−0.06	0.22	−0.24	−0.06	0.10	−0.26	0.14	−0.14	0.01	0.10	−0.04
	Bland-Altman -*p* value	0.64	0.62	0.18	0.09	0.47	0.44	0.11	0.33	0.08	0.92	0.54	−0.28
	Bias	0.02	0.02	0.06	−0.02	−0.00	0.06	−0.08	−0.02	0.02	0.08	−0.02	0.06
	ICC	0.96	0.83	0.94	0.96	0.96	0.83	0.90	0.98	0.96	0.90	0.92	0.96
FEF25-75	*r*	0.82	0.76	0.71	0.89	0.93	0.87	0.90	0.97	0.82	0.76	0.74	0.90
	*p* value correlation	<0.01	<0.01	<0.01	<0.01	<0.01	<0.01	<0.01	<0.01	<0.01	<0.01	<0.01	<0.01
	Differences	0.0 ± 0.6	0.0 ± 0.5	−0.1 ± 0.9	−0.1 ± 0.5	0.1 ± 0.4	0.1 ± 0.4	0.2 ± 0.5	0.0 ± 0.3	0.1 ± 0.5	0.0 ± 0.6	0.1 ± 0.8	−0.1 ± 0.5
	Bland-Altman - ρ	0.10	0.17	0.15	−0.08	0.06	−0.06	0.11	0.04	0.14	0.19	0.11	0.02
	Bland-Altman -*p* value	0.22	0.18	0.36	0.58	0.44	0.61	0.50	0.79	0.08	0.13	0.51	0.92
	Bias	−0.03	0.03	−0.05	−0.10	0.08	0.37	0.18	0.28	0.09	0.04	0.13	−0.09
	ICC	0.90	0.86	0.83	0.94	0.96	0.93	0.95	0.98	0.90	0.86	0.85	0.95
FEV1/FVC	*r*	0.71	0.63	0.59	0.79	0.78	0.78	0.60	0.85	0.73	0.69	0.47	0.85
	*p* value correlation	<0.01	<0.01	<0.01	<0.01	<0.01	<0.01	<0.01	<0.01	<0.01	<0.01	<0.01	<0.01
	Differences	0.0 ± 0.1	0.0 ± 0.1	0.0 ± 0.1	0.0 ± 0.1	0.0 ± 0.1	0.0 ± 0.1	0.0 ± 0.1	0.0 ± 0.1	0.0 ± 0.1	0.0 ± 0.1	0.0 ± 0.1	0.0 ± 0.1
	Bland-Altman - ρ	−0.02	−0.14	−0.19	0.26	−0.03	−0.05	0.11	−0.08	−0.10	−0.24	−0.23	0.18
	Bland-Altman -*p* value	0.78	0.26	0.26	0.08	0.71	0.70	0.50	0.59	0.24	0.06	0.17	0.22
	Bias	0.00	0.00	0.01	−0.02	0.01	0.01	0.03	0.06	0.01	0.01	0.04	−0.01
	ICC	0.83	0.76	0.72	0.88	0.88	0.87	0.75	0.92	0.84	0.81	0.62	0.92

Intra-methods agreement was assessed between the results of the two home spirometry results closest to the conventional spirometry (*i.e.*; Mes 1 and Mes 2). Inter-methods agreement was assessed between the results of the home spirometry results closest to the conventional spirometry (*i.e.;* Mes 1) and conventional spirometry results. Agreement over time was assessed between the results of the second closest home spirometry results to the conventional spirometry (*i.e.*; Mes 2) and conventional spirometry results. Agreement was assessed using Pearson correlation tests (coefficient of correlation *r* and *p* values are presented), Bland-Altman tests (Spearman coefficient of correlation ρ between the average and *p* values are presented), and by determining the intraclass correlation between results of the two spirometry.

### Real-life variability over time of home spirometry

Using all data collected from home spirometry, we then assessed its variability over time for FEV1, FVC, FEF25-75 and FEV1/FVC in the whole population and in each age groups and the results are presented in Table 3. In the whole population, and for each group there was more important variability over time in ascending order for FEV1/FVC, FVC, FEV1 and FEF25-75 as indicated by their normalized *Δ*max, coefficient of variation and normalized average variability (Table 3). We also demonstrated that variability over time was significantly higher in children than in the two other groups as indicated by significant higher normalized *Δ*max, coefficient of variation and normalized average variability (Table 3). However, except for both FEV1 and FVC normalized *Δ*max increased in teenagers compared to adults, and there was no significant difference according to variability over time between teenagers and adults (Table 3).

**Table 3:** Home spirometry variability over time.

Characteristics		All	Children	Teenagers	Adults	*p*
FEV1	Norm. *Δ*max (%)	49.6 [28.6; 68.1]	61.3 [48.5; 88.8]	49.5 [30.5; 71.3]	26.6 [21.0; 39.4]	<0.001^a,b,c^
	CoV (%)	11.5 [7.7; 16.1]	13.4 [10.6; 19.3]	10.7 [7.3; 14.3]	8.1 [5.9; 12.6]	<0.001^a,b^
	Norm. average variability (%)	8.9 [6.3; 12.9]	10.5 [8.4; 15.7]	7.6 [5.7; 10.0]	7.4 [5.1; 9.7]	<0.001^a,b^
FVC	Norm. *Δ*max (%)	44.9 [27.7; 65.2]	59.0 [42.5; 78.4]	43.1 [27.1; 65.2]	25.5 [18.7; 40.6]	<0.001^a,b,c^
	CoV (%)	10.0 [6.7; 14.5]	11.5 [8.8; 18.2]	9.3 [5.8; 14.3]	7.3 [5.3; 11.6]	<0.001^a,b^
	Norm. average variability (%)	8.1 [6.2; 11.6]	9.3 [7.3; 13.0]	7.0 [5.3; 9.9]	7.2 [5.1; 10.1]	<0.001^a,b^
FEF25-75	Norm. *Δ*max (%)	92.3 [58.0; 136.4]	131.8 [91.5; 171.6]	89.5 [58.5; 115.2]	61.5 [36.5; 80.3]	<0.001^a,b^
	CoV (%)	21.2 [15.1; 30.8]	28.0 [20.0; 36.1]	21.1 [14.0; 26.6]	16.0 [12.0; 21.6]	<0.001^a,b^
	Norm. average variability (%)	18.7 [13.4; 25.9]	23.9 [18.0; 29.8]	16.7 [11.8; 21.0]	14.4 [11.0; 18.8]	<0.001^a,b^
FEV1/FVC	Norm. *Δ*max (%)	34.7 [20.0; 53.5]	43.2 [28.7; 68.1]	34.2 [14.6; 53.9]	23.9 [13.4; 36.4]	<0.001^a,b^
	CoV (%)	8.0 [4.8; 11.8]	9.6 [6.4; 12.9]	7.2 [4.2; 10.8]	6.1 [4.3; 9.0]	0.001^a,b^
	Norm. average variability (%)	6.5 [4.7; 10.3]	8.7 [5.9; 11.5]	5.9 [3.6; 9.7]	5.8 [4.1; 8.0]	0.001^a,b^

Results are presented as medians with interquartile ranges [median (IQR25; IQR75)]. Comparison between groups were performed using the Kruskal-Wallis test with a Dunn post-test. *p* < 0.05 was considered significant. a Children vs. teenager; b Children vs. adults; c teenagers vs. adults.

FEV1, forced expiratory volume in one second; FVC, force vital capacity; FEF25-75, forced expiratory flow between 25% and 75% of vital capacity; *Δ*max, difference between the highest and the lowest values recorded; Norm., normalized; CoV, coefficient of variation.

## Discussion

Taken together, our results demonstrate good adherence and reliability of home spirometry in our population of PwCF with better adherence over time in children compared to teenagers and adults but higher reliability and lower variability over time in these last two groups compared with the former group.

Previous studies suggest that home spirometers are well accepted, easy to use and not burdensome for patients in various chronic diseases (19, 20) as well as in CF (14, 15). These studies reported good adherence to home spirometry but also highlighted a significant decrease over time (14, 15, 19, 20) as in the present study. We herein also demonstrated that children and teenagers use their device more frequently compared to adults with a higher percentage of patients performing weekly home spirometry and were less likely to stop using the device early. We also identified that after a month, adherence to spirometry remained stable in patients still using the device with a number of tests performed corresponding to 50%–60% of the expected objective (*i.e.,;* 3 per week for children/teenagers and 1 per week for adults corresponding to 78 and 26 tests for children/teenagers and adults respectively during the whole follow-up period) in the three groups of patients in agreement with adherence reported by a previous study (18). Since no previous study compared adherence to home spirometry between different age groups, the difference in adherence between children, teenagers and adults highlighted in our study was not surprising. Indeed, a study including adult patients report lower adherence (20) than those including children and or teenagers (14). Several factors may have influenced the difference in patients' adherence between groups. First, recommendations were different for the two groups, since at least 3 weekly measurements were required for children compared to only one in adults leading to an increased number of tests performed in children and teenagers compared to adults. Secondly, the difference in adherence between groups might be due to the encouragement of physiotherapists who were seeing patients for regular treatment more frequently in children and teenagers than in adults. Indeed, the presence of a caregiver is known to promote compliance in chronic respiratory diseases (21) and this can explain the difference in adherence and early stops between adults and children/teenagers. In addition, children probably have more parental support than teenagers who are more independent. Thirdly, the disease severity, greater in adults, may impact adherence due to increased daily medication and reduced quality of life. The addition of an extra device may have made daily life more difficult, discouraging them from using this new device (17).

In addition to good adherence, home spirometry demonstrated good to excellent reliability compared to conventional spirometry in our population in agreement with previous studies (14, 15) and despite unsupervised use by a trained technician. However, we identified better reliability and a lower variability in adults and teenagers than in children regarding FEV1, FVC, FEF25-75 and FEV1/FVC as reported for conventional spirometry (22, 23). This increased variability in children has previously been reported for both home spirometry (24) and conventional spirometry (25) and may be related to greater difficulties for children to perform forced spirometry. The association between age and lung function variability should be considered when interpreting home spirometry in longitudinal analyses.

Our results indicate that home spirometry can be useful to follow more precisely the lung function over time and could be used to detect exacerbations at an early stage and thus change the management of the disease (decreased clinic attendances and/or number of unplanned reviews). However further studies are needed to confirm this. Indeed, previous studies aimed to detect early and treat CF exacerbations using home telemonitoring to prevent lung function decline (18, 26). However, the study by Lechtzin et al*.* using a variability of more than 10% of FEV1 from baseline as a threshold for exacerbation detection was able to accurately detect exacerbations in adolescent and adults but without improvement in lung function decline (18). Van Horck et al*.*, demonstrated that exacerbations can accurately be predicted using a linear mixed model including both mean FEV1 and a respiratory symptoms in children with CF (26). However in their study they did not identify significant FEV1 variability in the weeks prior to exacerbation, thus predicting exacerbation using their model did not result in FEV1 change but more in the severity of the disease (26). Moreover, to accurately predict exacerbations, the model included a mean FEV1 lower than 77% of predicted values, representing a significant impairment of FEV1 in children (26). Thus, identifying models predicting very early the occurrence of exacerbation remains necessary and would require as covariate the variability of the function and the age of the patients.

Several limitations must be pointed out. Firstly, home spirometry data must be transmitted by the patients and some tests may not have been transmitted which can lead to biases in observance and variability analyses. Secondly, the impact of this new device on patients' quality of life and the collection of reasons for discontinuation were not evaluated in this study and could have made it possible to identify ways of improving patient adherence. Third, our study is a single-centre study, and the local management of PwCF could have influenced the adherence results of children and adults. However, the Hospital Center of Bordeaux is involved in the management of most PwCF patients in southwestern France. Moreover, the size of our population represented one of the largest cohorts assessing observance, reliability and variability of home spirometry in CF patients which allowed for the age-group analysis. Fourth, the fact that the follow-up period was limited to six months has decreased our analysis to a short-term reliability of home spirometry compared to conventional spirometry. However, after 6 months, adherence remained good in children and adolescents, but nearly 50% of adult patients had already been lost indicated that long term acceptability can be more of an issue in adults. A follow-up of 12 months or more would have been useful to determine the long-term adherence of patients and the reliability of home spirometry. Nevertheless, our results indicated good agreement, improving with the age of the patient, between home and conventional spirometry three months after the first use of the device introduction.

## Conclusion

We have demonstrated the real-life efficacy of home lung function telemonitoring in providing reliable data with good adherence in a significant proportion of patients, but further studies are now needed to demonstrate its usefulness to prevent and/or early detect acute exacerbations for which earlier intervention might be of greater benefit.

## Data Availability

The raw data supporting the conclusions of this article will be made available by the authors, without undue reservation.
